# A novel hierarchical porous nitrogen-doped carbon derived from bamboo shoot for high performance supercapacitor

**DOI:** 10.1038/s41598-017-06730-x

**Published:** 2017-08-04

**Authors:** Xiufang Chen, Junyi Zhang, Bo Zhang, Shanmu Dong, Xingcui Guo, Xindong Mu, Benhua Fei

**Affiliations:** 1 International Centre for Bamboo and Rattan, Beijing, 100102 P.R. China; 2 Key Laboratory of Bio-based Materials, Qingdao Institute of Bioenergy and Bioprocess Technology, Chinese Academy of Sciences, Qingdao, 266101 P.R. China

## Abstract

Porous N-doped carbons hold good prospects for application in supercapacitor due to their low-cost, large surface area, good surface wettability, high electrical conductivity as well as extra pseudocapacitance. However, most synthetic methods required the tedious and multiple-step process with the assistance of hard/soft templates or the massive use of chemical reagents, and exogenous nitrogen sources, which made them difficult to realize industrial production and application. Here, we described a novel hierarchical porous N-doped carbons fabricated by a facile and sustainable approach via hydrothermal treatment and subsequent carbonization process by using renewable bamboo shoots as the starting material without any templates, additional chemical activation and nitrogen source. The obtained bamboo shoot-derived carbons possessed a large BET surface area (up to 972 m^2^ g^−1^), hierarchically interconnected porous framework, rich and uniform nitrogen incorporation (3.0 at%). Benefiting from these unique features, the novel carbon-based electrode materials displayed a high capacitance of 412 F g^−1^ in KOH electrolyte and long cycling life stability. Thus, an advanced electrode material for high-performance supercapacitor was successfully assembled by a simple and scalable synthesis route with abundant renewable resources freely available in nature.

## Introduction

To face the challenge of the finite nature of fossil fuels and global warming, there is an urgent need for the development of energy storage devices with high power density to store energy and supply electricity^1, 2^. Because of prominent features such as long cycling life, high power density and fast charging capability, supercapacitor is recognized as an ideal energy storage candidate for the application in electric vehicles and portable electronics^3^. Unlike lithium-ion batteries with relatively low power density (<1 kW kg^−1^), supercapacitor could offer high power density (1–10 kW kg^−1^)^4^. Moreover, supercapacitor could store charge by highly reversible adsorption/desorption of electrolyte ions from the surface of the electroactive materials or speeding up redox reactions, which facilitates rapid energy capture and delivery^5^. Nevertheless, the large-scale industrial application of supercapacitor is often restricted by the relatively low capacitance.

As the electrode materials are the key to determine the electrochemical performance of supercapacitor, the development of high-performance electrode materials is essential for the practical use of supercapacitors. To improve the electrochemical performance, various electrode materials have been designed and constructed^6–9^. Among them, carbon-based materials have drawn significant attention as promising electrode materials for supercapacitors due to the advantages of low cost, easy availability, high surface area, high conductivity and good stability^10–12^. So far, traditional activated carbons have been used widely as the dominant electrode materials for commercial supercapacitor, but suffers from low energy density and finite charge-discharge performance, which considerably restrict its further application^13^. Therefore, much effort has been devoted to developing novel nanoporous carbon materials to solve the problems^14–16^. A promising strategy is to dope heteroatoms (N, O, S, B) into nanoporous carbon materials to improve the capacitance performance^17–19^. Particularly, porous N-doped carbon materials have been proven as superior electrode materials for supercapacitor. Owing to the higher electronegativity of nitrogen than carbon, the incorporation of nitrogen into carbon frameworks increases the positive charge density of carbon, which is conductive to the enhancement of surface wettability, surface polarity, electrical conductivity as well as extra pseudocapacitance^20^. Furthermore, highly porous structure with a large surface area in electrode materials would also facilitate the access of the electrolyte ions and provide abundant electroactive sites for efficient charge adsorption. The supercapacitors with such electrode materials would combine the electrical double-layer capacitor (EDLC) with pseudocapacitance to achieve good long-term cycling stability, relatively high specific capacitance and rapid charge-discharge process. Current technologies for porous N-doped carbon fabrication are commonly based on two strategies^21, 22^: post-treatment of porous carbon with nitrogen source, or *in-situ* doping by carbonization of nitrogen-rich precursors. These methods either required multi-step process, or the assistance of hard/soft templates, or the chemical activation with a large amount of reagents, or the strict control of synthesis conditions^22, 23^, which limits its mass production. It is therefore highly desirable to fabricate porous N-doped carbon with a large surface area and porous microstructure for high-performance supercapacitor with renewable materials by a simple, cheap, green and template-free process.

Biomass-derived porous carbon materials have recently attracted considerable attention due to their abundance, low-cost, readily availability, rapid regeneration and environmental friendliness. In particular, some nitrogen-enriched biomass is a kind of desirable starting materials as both carbon and nitrogen source to produce N-doped carbon materials. Recently, crude biomass such as fungi^24^, starch^25^, egg white^20^, corn grain^26^, celtuce leaves^27^, watermelon^28^, sheep manure^29^ and lignocellulosic materials^30^ were used as raw materials to produce porous carbons, which exhibited good performance in solid adsorbents for CO_2_ capture or electrode materials for supercapacitors. To date, the chemical activation of biomass by alkali (e.g. KOH, NaOH) was still the preferred method to produce porous carbons with a large specific surface area and high porosity. While some progress have been made towards the fabrication of N-doped carbons derived from biomass as the starting materials, it still remains challenges to directly utilize the crude biomass via a cheap and sustainable synthetic process.

In this work, a low-cost, green, simple and easily scalable method was explored to fabricate porous N-doped carbons with bamboo shoots as raw materials. Bamboo is recognized as the fastest-growing woody, evergreen and perennial plant, which is one of the richest resources in China. Bamboo shoots, known as the bud of bamboo, were commonly used as a traditional forest vegetable in China for over 2000 years^31^. It widely distributes from plains to hills lower than 1400 m, which could be freely available from nature. The easily available bamboo shoots contain abundant cellulose, hemicellulose, lignin, protein, amino acids, fats, carbohydrates, etc^32^. Particularly, the bamboo shoots are rich in nitrogen-containing organics such as protein, amino acids, which could be ideal carbon and nitrogen sources to produce N-doped carbons^31^. Here, a range of porous N-doped carbon materials was produced by a hydrothermal treatment and subsequent carbonization process in inert atmosphere without using any templates or additional chemical activation. The obtained materials possessed a large BET surface area with extensively porous structure and uniform nitrogen dopant distribution. This novel N-doped carbon material was found to be an excellent electrode for supercapacitors with superior electrochemical performance.

## Results and Discussion

### Fabrication of nanostructured N-doped carbons

**Figure 1:**
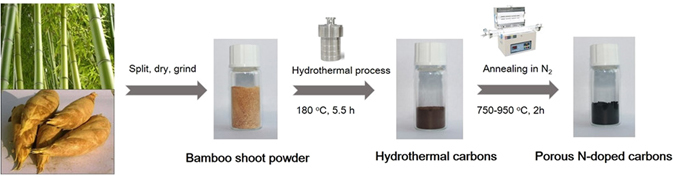
Schematic for the preparation of the porous N-doped carbon materials.

A facile and green synthetic route was developed to prepare nanostructured N-doped carbons using naturally available crude biomass bamboo shoots as both C and N sources without using any templates or chemical reagents for activations. It is well-known that the bamboo shoots have some unique characteristics in chemical compositions and physical textures, which significantly differ from the mature bamboo and other biomass sources^32^. The lignin, crystalline cellulose and xylan predominantly accumulate in the cell wall in the bamboo shoot, which is unlike high lignification of the mature bamboo. The low degree of lignification in bamboo shoots along with considerable amounts of acid-soluble lignin such as *p*-coumaric acid and ferulic acid make them with unlignified and loose tissues and less extended crystalline cellulose in textures, which are beneficial for the permeation of water molecules into their tissues and in turn permit the readily decomposition into small carbon nanoparticles during the hydrothermal process^33^. Apart from the three components of biomass e.g. cellulose, hemicellulose and lignin, the existence of quite amount of sugars, amines and proteins in the bamboo shoots also facilitate them to be transformed into spherical-like carbon particles^32, 34–36^. More importantly, bamboo shoots are intrinsically rich in nitrogen-containing organics including protein and amino acids. The nitrogen content can reach up to 4.3% based on the dry weight of bamboo shoot, which allow the feasibility of *in-situ* incorporation of nitrogen in the carbon matrix due to the reactions between the as-formed carbon intermediates with nitrogen-containing organics during carbonization process. As such, the overall fabrication process of N-doped carbons involves a facile hydrothermal treatment at 180 °C and post-carbonization at 750–950 °C under N_2_ atmosphere, as shown in Fig. 1. Briefly, the bamboo shoots gathered from the bamboo forest, were cut into slices, dried and ground into powders followed by the hydrothermal process in a Teflon-inner stainless steel autoclave. In this process, the bamboo shoots were depolymerized and hydrolyzed followed by dehydration, retro-aldol condensation and aromatization under dilute acidic conditions and self-generated pressure to yield dark brown hydrochars^37, 38^. Subsequently, the resulting hydrochars were filtered, washed thoroughly with water to remove the soluble impurities and then dried under air. At last, the obtained solid hydrochars were calcined at 750–950 °C under N_2_ atmosphere to decompose unstable compositions to form a more-stable structure and generate porosity, and remove oxygenated groups from the surface to enhance the electronic conductivity^39^. The as-prepared materials were denoted as BFH-T, where T represents the calcination temperature. For comparison, the material derived from the dried bamboo shoots calcined at 850 °C under nitrogen atmosphere without hydrothermal treatment process was also prepared accordingly, denoted as BF-850.

### Characterization of nanostructured N-doped carbons

**Figure 2:**
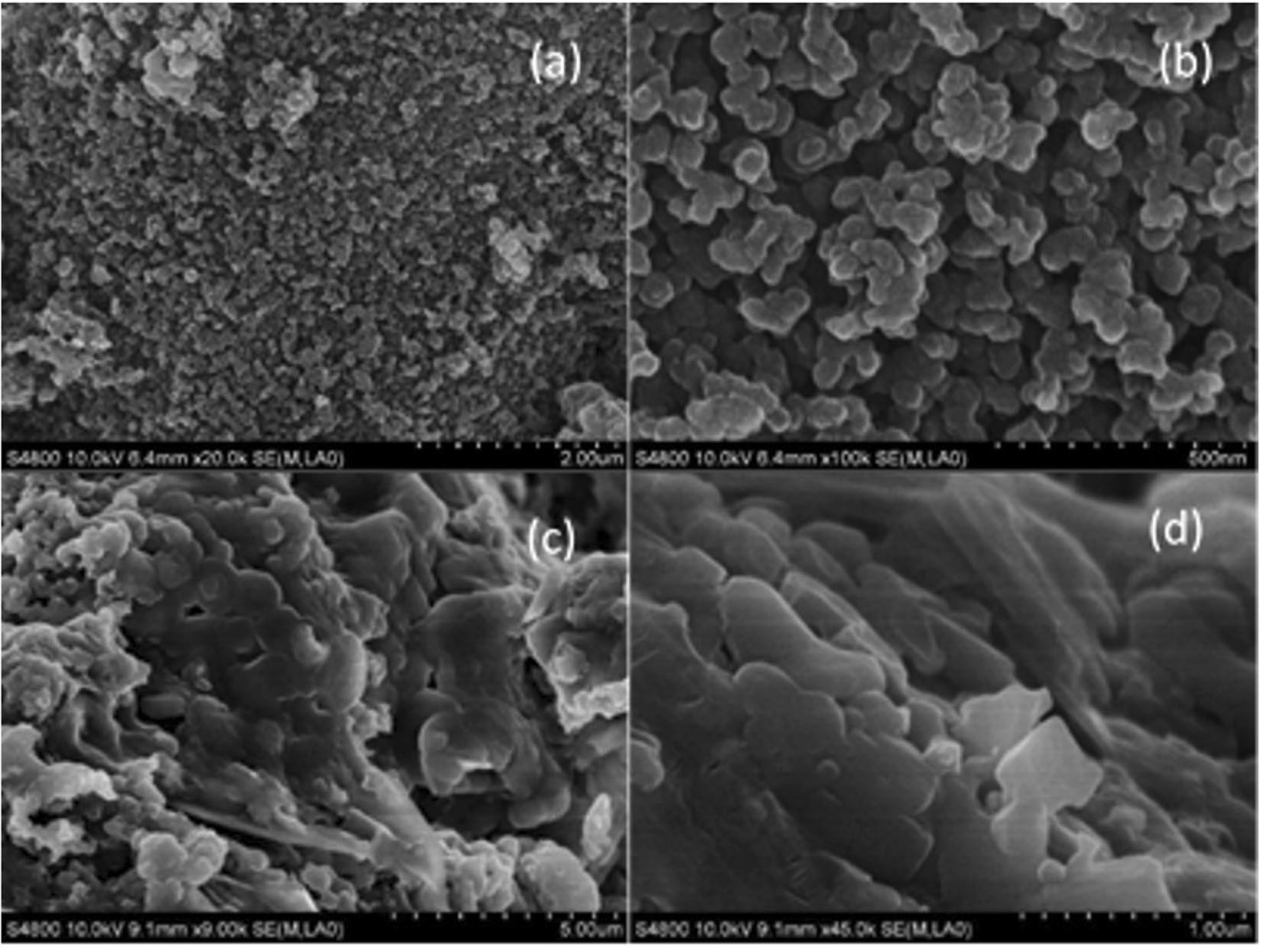
SEM images of BFH-850 (**a**,**b**) and BF-850 (**c**,**d**).

**Figure 3:**
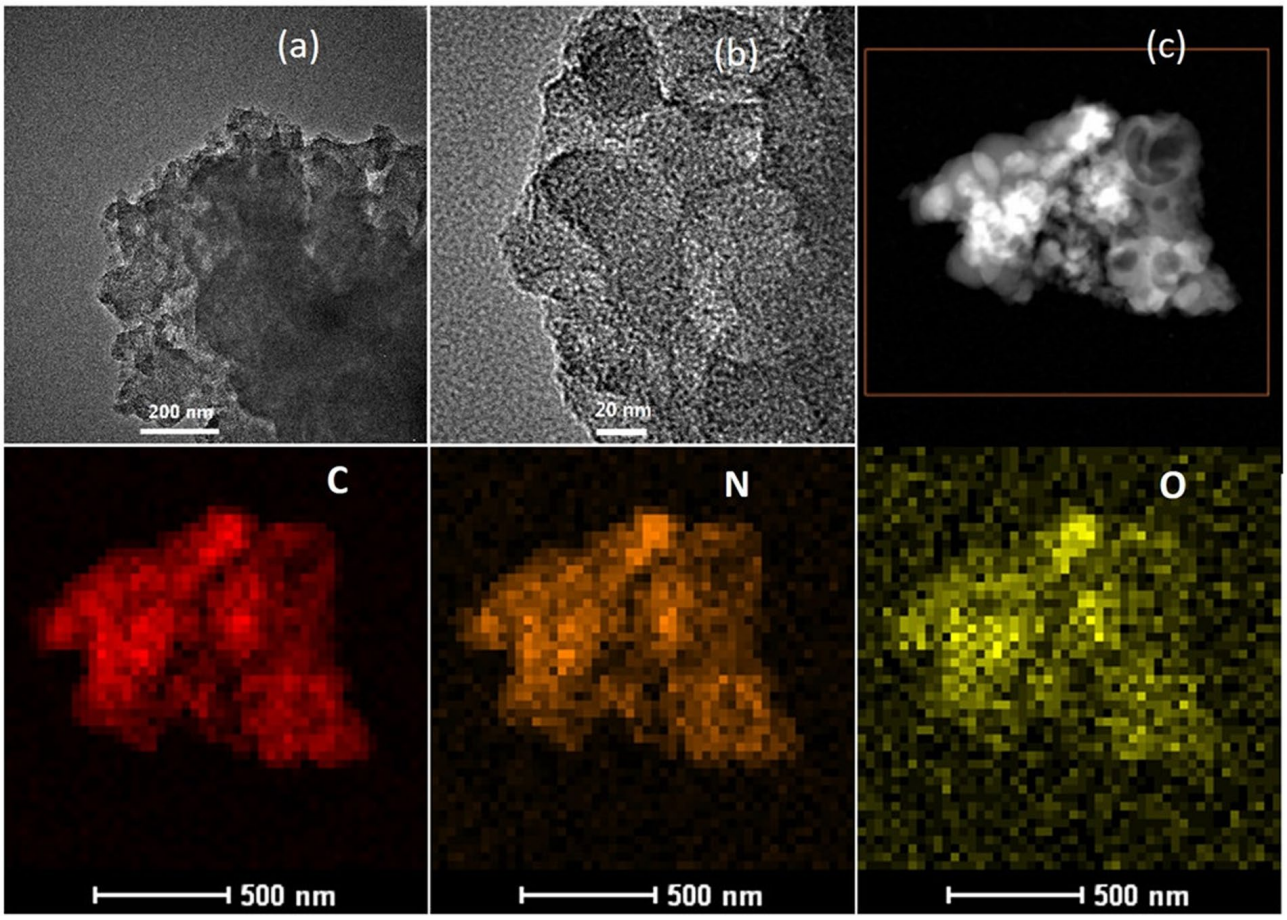
Representative TEM images of BFH-850 (**a**,**b**), HAADF-STEM image (**c**) and corresponding mapping of carbon, nitrogen and oxygen of BFH-850.

The morphology and microstructure of the bamboo shoot-derived carbon materials were analyzed by SEM and TEM measurements. Figure 2 displayed the representative SEM images of BFH-850, with BF-850 as a reference. It was obvious that microstructure for BFH-850 and BF-850. BFH-850 (Fig. 2a,b) exhibited a loose and interconnected structure with interstitial porosity, which was made up of numerous uniformly-distributed spherical-like nanoparticles with small sizes of about 40–80 nm. For comparison, BF-850, prepared by direct carbonization at high temperature in inert gas, showed a relative close-packed structure with many irregular fused sphere-like particles (Fig. 2c,d). These differences implied that hydrothermal treatment process was essential for forming and stabilizing well-distributed spherical-like nanoparticles. TEM and HRTEM analysis in Fig. 3a,b illustrated the existence of spherical-like carbon nanoparticles with hierarchical porous structure in the BFH-850 sample. STEM-EDX was also employed to investigate the elemental composition and distribution of BFH-850. The results, shown in Fig. 3, indicated that BFH-850 mainly consisted of carbon, nitrogen and oxygen elements with perfectly homogeneous distribution of these elements. The elemental analysis results further supported the presence of nitrogen element in the bamboo shoot-derived carbon materials. The nitrogen content based on elemental analysis (shown in Table 1) were about 3.0%, 2.9%, 1.4% and 2.5% for BFH-750, BFH-850, BFH-950 and BF-850, respectively. These results confirmed that the bamboo shoot-derived carbon materials fabricated by hydrothermal treatment and post-carbonization were N-doped carbon materials, which were composed of a large number of spherical-like nanoparticles with nanoporous structure and uniform incorporation of nitrogen. As expected, there was a gradual decrease of nitrogen content with increasing carbonization temperature, as nitrogen species were further removed by reaction with oxygenated functions or formation of gaseous products (e.g. NO, N_2_ and NH_3_) when the carbon materials were carbonized at the higher temperature.

**Table 1:** Textural properties and chemical composition of carbon materials.

Sample	S_BET_^a^ (m^2^ g^−1^)	V_total_^b^ (cm^3^ g^−1^)	V_micro_^c^ (cm^3^ g^−1^)	C^d^ (at%)	N^d^ (at%)	Surface O^e^ (at%)	Surface N^e^ (at%)
bamboo shoot	0.4	0.002	/	41.7	4.27	/	/
BFH^f^	45	0.039	/	/	/	/	/
BF-850	264	0.130	0.127	72.3	2.51	/	/
BFH-750	913	0.622	0.288	74.2	2.96	12.6	2.6
BFH-850	972	0.619	0.335	73.4	2.86	13.0	2.4
BFH-950	624	0.407	0.214	78.2	1.42	9.9	1.2

^a^BET surface area calculated from the linear part of the BET plot (P/P_0_ = 0.1-0.2). ^b^Total pore volume, taken from the volume of N_2_ adsorbed at P/P_0_ = 0.99. ^c^t-plot micropore volume. ^d^Calculated from the results of elemental analysis. ^e^Calculated from the results of XPS spectra. ^f^the hydrothermal carbons.

**Figure 4:**
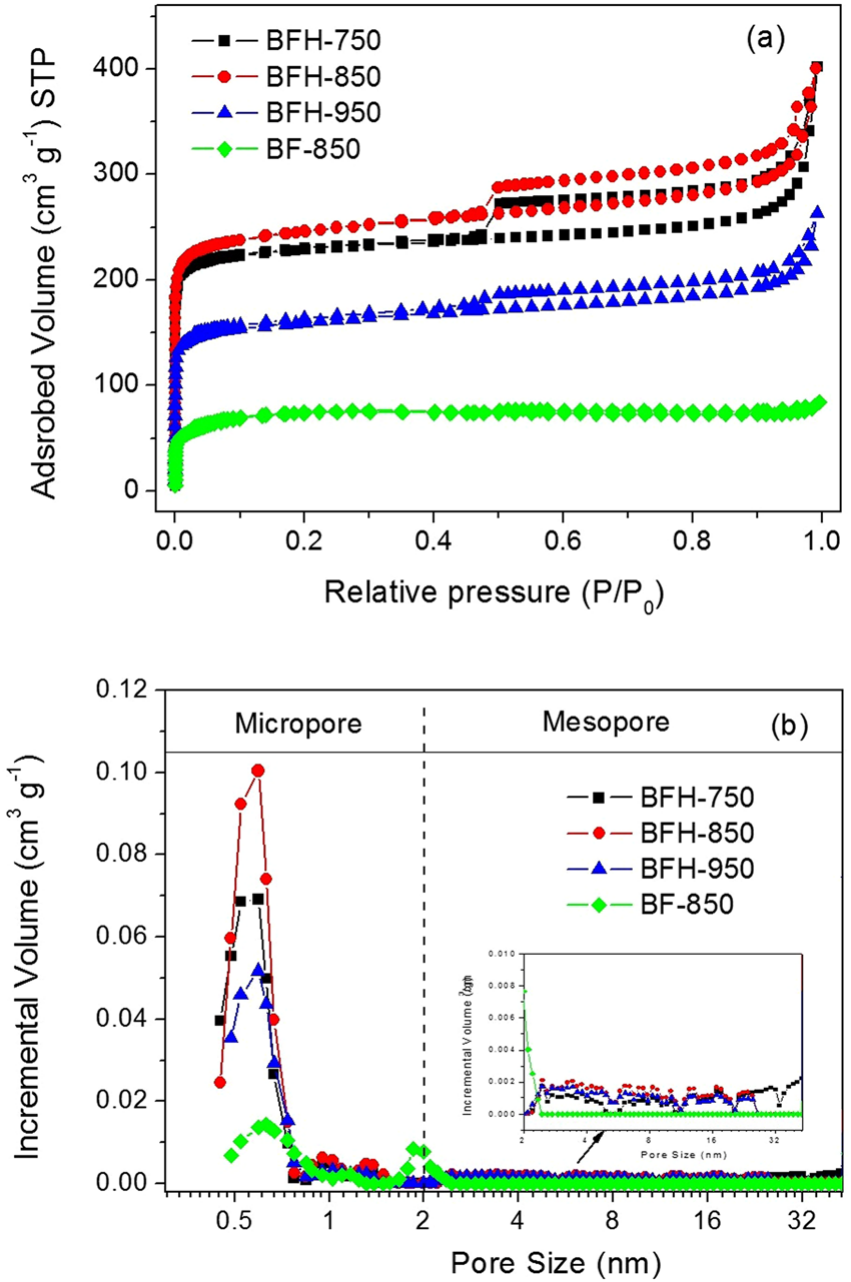
Nitrogen adsorption/desorption isotherm (**a**) and pore size distribution (**b**) of BF-850, BFH-750, BFH-850 and BFH-950.

To further study the influence of hydrothermal treatment and carbonization temperature on the porous texture of bamboo shoot-derived carbon materials, N_2_ sorption measurement at 77 K were carried out. Fig. 4a exhibited the N_2_ adsorption/desorption isotherms of BFH-750, BFH-850 and BFH-950, with BF-850 as a comparison. All the samples showed significant nitrogen gas adsorption at low pressure (<0.1), suggesting the existence of a large amount of micropores in the bamboo shoot-derived carbon materials. For BFH-750, BFH-850 and BFH-950 samples, the isotherms were all close to type IV with H1 hysteresis loop^21, 40, 41^, reflecting the mesoporous structure were also formed by hydrothermal treatment and subsequent carbonization process. Besides, there was a certain amount of nitrogen gas adsorption at the relatively high pressure (>0.8), indicating a large external surface area. In general, the total amount of nitrogen gas adsorption were greatly influenced by the carbonization temperature, which followed the tread of BFH-850 > BFH-750 > BFH-950. For comparison, BF-850 showed typical type I isotherm with little hysteresis loop, which was indicative of microporous material. The porous structure was also supported by the pore distributions shown in Fig. 4b. The samples of BFH-750, BFH-850 and BFH-950 primarily contained micropores with pore size of 0.5 nm, 0.9 nm and 1.3 nm, and some mesopores with pore size in the range of 2 nm to 50 nm (see inset in Fig. 4b). However, BF-850 is mainly micropores with pore size of ∼0.6 nm, 1.1 nm and 1.9 nm.

The results of surface area and pore volume were summarized in Table 1. Obviously, the surface area of BFH samples (972 m^2^ g^−1^ for BFH-850) was much higher than that of BF-850 (264 m^2^ g^−1^), supporting that hydrothermal treatment played an important role in the formation of porous structure in the carbon materials. The calculated surface area increased slightly from 913 m^2^ g^−1^ for BFH-750 to 972 m^2^ g^−1^ for BFH-850, and decreased obviously to 624 m^2^ g^−1^ for BFH-950, while the total volume reduced from 0.622 cm^3^ g^−1^ for BFH-750 and 0.619 cm^3^ g^−1^ for BFH-850 to 0.407 cm^3^ g^−1^ for BFH-950. These results confirmed that carbonization temperature was also a significant factor for the pore structure. Notably, the decrease of surface area and porous volume for BFH-950 might be due to the collapse of porous structure when increasing carbonization temperature up to 950 °C. Unlike BF-850 mainly composed of microporosity (98%, 0.127 cm^3^ g^−1^), BFH-850 had a distinct micro-mesopore size distribution (V_micro_ ∼ 0.335 cm^3^ g^−1^, V_meso_ ∼ 0.284 cm^3^ g^−1^). The results suggested that hydrothermal treatment greatly enhanced both the micropore and mesopore volumes. The formation of abundant porosity in the framework was speculated to associate with the decomposition of unstable compositions to generate gaseous species (e.g. CO, CO_2_, NO, N_2_, and NH_3_) during carbonization process^21^. As hydrothermal process promoted the formation of many spherical-like nanoparticles with small size and interstitial porosity, the interparticle pores between primary carbon particles also contributed to the mesoporosity^35^. It was known that different pore type displayed different roles in the supercapacitor performance^42^. A large amount of micropores could offer sufficient space to allow access to the electrolyte ions transportion, which were basic to the high energy storage. The existence of mesopores would accelerate the kinetic process of ion diffusion and help to ameliorate the power performance at high current densities. Thus, such hierarchical porous nanomaterials, which facilitated electrolyte penetration and ion diffusion, are more ideal for the high-performance electrode for supercapacitors.

The crystallographic structures of the bamboo shoot-derived carbon materials were investigated by XRD. As shown in Fig. S1, all the samples displayed two broad and weak characteristic graphitic carbon peaks at ∼24° and ∼44°, attributable to (002) and (101) planes of hexagonal graphite, respectively^21^. The broad and weak peaks implied the presence of amorphous carbons in the carbon materials. Beyond that, BF-850 had some other peaks of inorganic compounds such as KCl and KHCO_3_, which was originated from the minerals of the raw materials or the product formed during the carbonization. To further analyze the structures of bamboo shoot-derived carbons, Raman spectroscopy measurements were performed. Fig. S2 gave the Raman spectrum results of BFH-750, BFH-850, BFH-950 and BF-850 samples. All the samples possessed two typical peaks at 1341 cm^−1^ and 1590 cm^−1^, belonging to D band (disordering degree in the structure) and G band (graphitic order), respectively^33, 43^. The I_G_/I_D_ value was normally closely related to the level of graphitic ordering in the carbons. The I_G_/I_D_ values were 0.85, 0.86, 0.90 and 0.87 for BFH-750, BFH-850, BFH-950 and BF-850, respectively. These results confirmed the disordered structure in the bamboo shoot-derived carbon materials. With the rise of carbonization temperature, the carbon material became more ordered, which probably meant a better conductivity.

**Figure 5:**
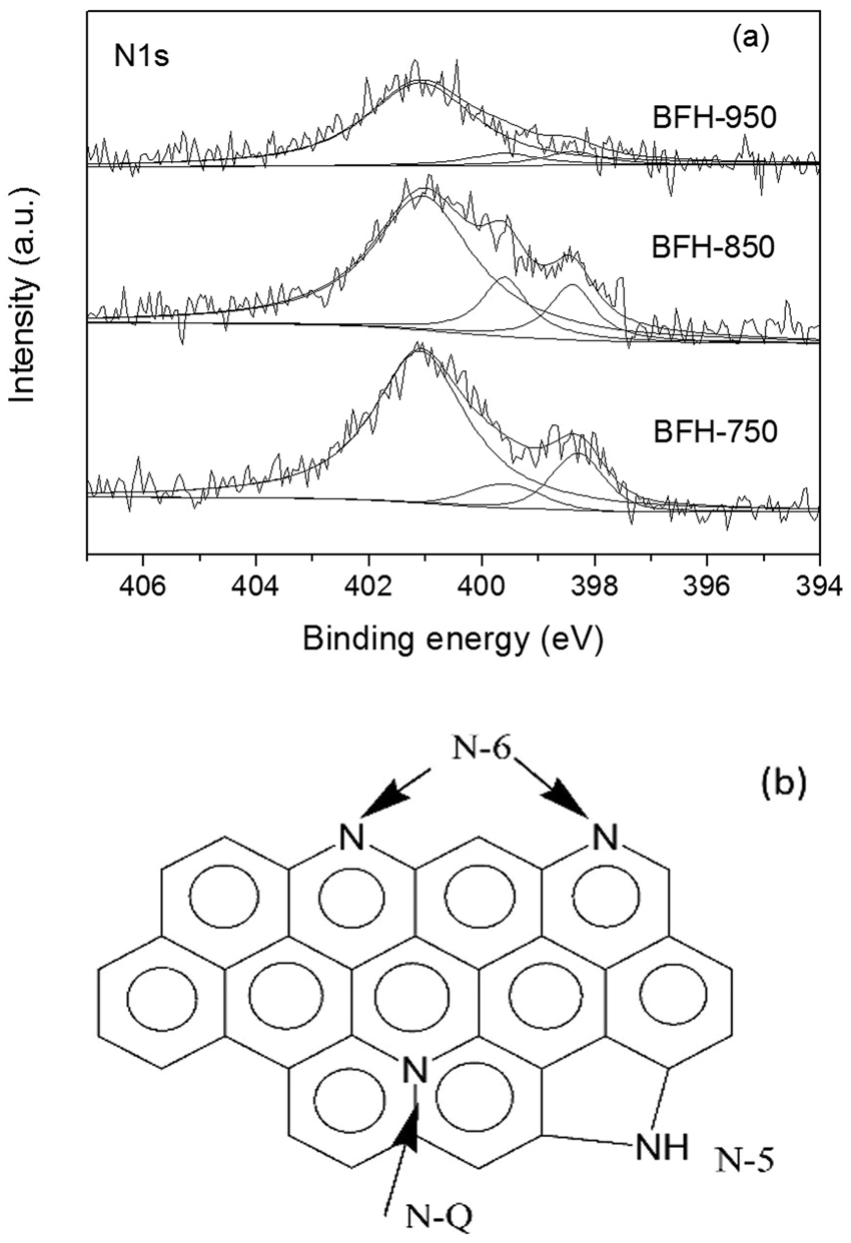
(**a**) XPS spectra of N1s for BFH-750, BFH-850 and BFH-950, (**b**) possible structure of N species doped on carbon.

The bamboo shoot-derived carbon materials were also subjected to XPS analysis to investigate the effect of carbonization temperature on the surface contents and chemical states. The XPS survey in Fig. S3 showed that carbon, nitrogen, oxygen, chlorine and silicon elements coexisted in the BFH-850 sample with content of 82.4 at%, 2.4 at%, 13.0 at%, 0.9 at% and 1.3 at%, respectively. The surface nitrogen content calculated by XPS measurement was in consistent with the elemental analysis result of 2.9%, supporting the uniform incorporation of nitrogen in the framework of carbons. It can be observed in Fig. 5a that N1s high-resolution spectra of BFH samples can be deconvoluted into a dominant peak at 401.1 eV and two small peaks at 399.6 eV and 398.4 eV. The peak at 401.1 eV was assigned to quaternary nitrogen (N-Q) and the two small peaks at 399.6 eV and 398.4 eV belonged to pyrrolic nitrogen (N-5) and pyridinic nitrogen (N-6), respectively (Fig. 5b)^44^. By comparing with the results of BFH-750, BFH-850 and BFH-950 summarized in Table 1, the surface nitrogen content was just slightly decreased from 2.6 at% for BFH-750 to 2.4 at% for BFH-850, but reduced remarkably to 1.2 at% for BFH-950. The oxygen content of the samples also had the similar variation tendency. A careful observation showed that the N-Q and N-6 species were predominant in these BFH samples. For BFH-850 sample, the proportions of N-Q, N-5 and N-6 in the BFH-850 were 78.7%, 8.5% and 12.8%, respectively. In general, the N-Q and N-6 in the carbons were regarded as electroactive sites, which would benefit the enhancement of electrical conductivity as well as capacitances. These results were also supported by FT-IR spectra shown in Fig. S4, which confirmed the presence of the amino groups, carbonyl group and hydroxyl group on the surface of the carbon materials, and the content of both nitrogen and oxygen-rich functional groups were reduced progressively after they were carbonized at higher temperature. The results were in good agreement with those of elemental analysis, suggesting that during carbonization process at high temperature, some nitrogen species and oxygen species were escaped by formation of gaseous products (e.g. CO, CO_2_, NO, N_2_ and NH_3_). Note that the carbonization of N-doped carbons at temperature above 850 °C caused a distinct decrease of surface area, collapse of pore structure and loss of nitrogen species, which were negative factors for the performance of supercapacitors. In the range of 750–850 °C, although carbonization caused a slight loss of nitrogen species, but increased the surface area, porosity and graphitization degree of carbons, which might favorable influence the electronic conductivity and electrochemical property of N-doped carbons. Based on the above analysis, BFH-850 sample was expected to be the most appealing material for the electrode for supercapacitors.

### Electrochemical performance of nanostructured N-doped carbons

**Figure 6:**
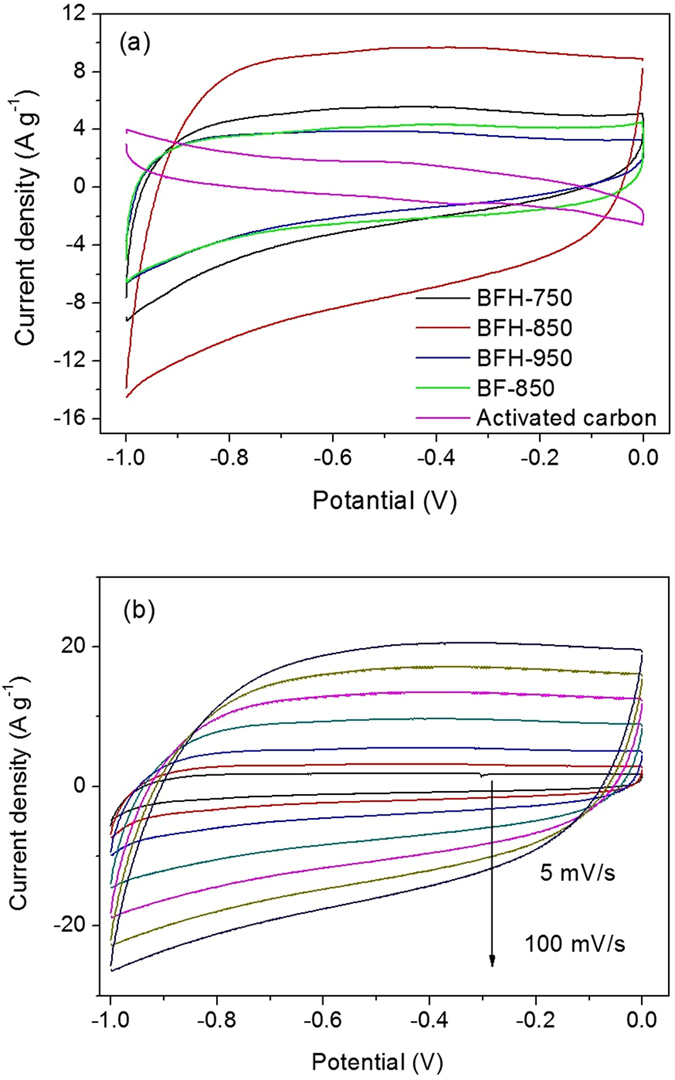
(**a**) CV curves of BF-850, BFH-750, BFH-850, and BFH-950 at a scan rate of 50 mV s^−1^, (**b**) CV curves of BFH-850 at various scan rates.

**Figure 7:**
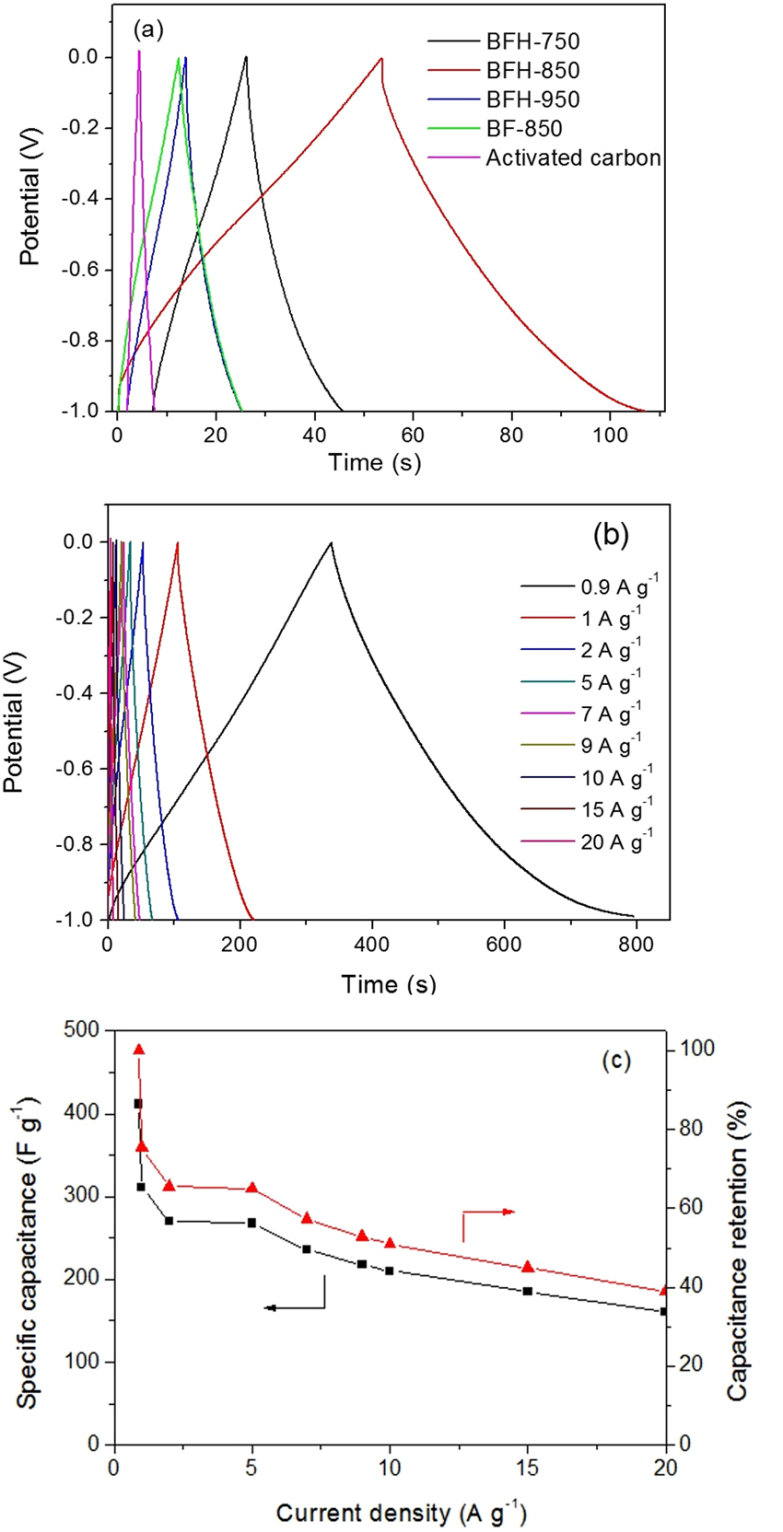
(**a**) Galvanostatic charge-discharge curves at a current density of 5 A g^−1^ for BF-850, BFH-750, BFH-850, and BFH-950, (**b**) Galvanostatic charge-discharge curves under various current densities for BFH-850, (**c**) Specific capacitance of BFH-850 at various current densities.

To validate the promising applications, the electrochemical performances of bamboo shoot-derived N-doped carbon materials were evaluated in a three-electrode system. Cyclic voltammetry (CV) and galvanostatic charge/discharge measurements were tested firstly to investigate their capacitive performances. The typical capacitive behavior of the BFH samples was shown in Fig. 6a. All BFHs electrode displayed quasi-rectangular features of the CV curves between −1 and 0 V at 50 mV s^−1^. A slight distortion generally caused by redox reactions was also observed in the CV curves, which might reflect the combination of double-layer and pseudocapacitance associated with nitrogen functionalities^24^. The capacitive performance was also found to be dependent on the carbonization temperature. The encircled area of the CV curve was increased rapidly with increasing carbonization temperature from 750 °C to 850 °C, but dropped abruptly with higher temperature. The results indicated that BFH-850 had the highest capacitance among the three samples, demonstrating that BFH-850 was the most promising electrode material for supercapacitors. As discussed above, all the factors including the largest efficient surface area, hierarchical meso/microporous structure, relatively high graphitization degree of carbons and rich nitrogen functionalization of BFH-850 material probably contributed to the highest electrochemical performance among the BFH materials carbonized from 750 °C to 950 °C. Moreover, the CV curves of BFH-850 kept a quasi-rectangular shape with the scan rate ranging from 5 mV s^−1^ to 100 mV s^−1^ (Fig. 6b), indicating good capacitance characteristics of bamboo shoot-derived carbons. For comparison, the CV performance of BF-850 and commercial activated carbon were also measured at the same condition (50 mV s^−1^), the encircled area of the CV curve for BF-850 and activated carbon were much smaller than BFH-850. As shown in Fig. 7a, the galvanostatic charge/discharge curves of bamboo shoot-derived carbons revealed a relative symmetrical triangle. The specific capacitance calculated by discharge curves at the current density of 5 A g^−1^ were 50, 270 and 58 F g^−1^ for BFH-750, BFH-850 and BFH-950, respectively, which was much higher than that of BF-850 (26 F g^−1^) and activated carbon (16 F g^−1^). The performances were in keeping with the trend of CV results. The galvanostatic charge/discharge curves of BFH-850 at various current densities were also measured and the results were shown in Fig. 7b,c. Excitingly, a high specific capacitances of 412 F g^−1^ were obtained at 0.9 A g^−1^. When increasing the current density to 5 A g^−1^, there was a decrease in the specific capacitance (270 F g^−1^) with good capacitance retention of 65.5%, indicating their good rate capability. Further increasing the current density to 20 A g^−1^, the specific capacitance was still maintained at 161 F g^−1^. It was also worthy to note that BFH-850 had a higher capacitive performance than those of most other biomass-derived carbon materials in KOH electrolyte (Table 2). More importantly, to achieve porous structure with large surface area, most crude biomass-derived carbons reported before were prepared by chemical activation, which not only needed complex, time-consuming process, but also caused serious environment pollutions. In our cases, the bamboo shoot-derived N-doped carbon materials with hierarchical pore structure and a high surface area were prepared by a facile hydrothermal treatment and subsequent carbonization without any templates, additional chemical activations and nitrogen sources, which could provide a good basis for the exploitation of the electrode materials for supercapacitor with cheap and mass production. The synthetic method had the advantages of simple, sustainable, low-cost and easy to produce in large scale.

**Table 2:** Comparison of electrochemical performance of biomass-derived carbon materials in a three-electrode system.

Precursor	S_BET_ (m^2^ g^−1^)	Activation method	Capacitance (F g^−1^)	Measurement condition	Ref
Auricularia	80	hydrothermal treatment + carbonization	196	6 M KOH 5 mV s^−1^	22
Celtuce leaves	3404	KOH	421	2 M KOH 0.5 A g^−1^	25
Banana peel	1650	Zinc complexes	206	6 M KOH 1 A g^−1^	47
Pomelo peel	2725	KOH	342	6 M KOH 0.2 A g^−1^	48
corn husk	928	KOH	356	6 M KOH 1 A g^−1^	49
shiitake mushrooms	2988	H_3_PO_4_ + KOH	306	6 M KOH 1 A g^−1^	50
fungus	1103	hydrothermal treatment in KOH solution + carbonization	374	6 M KOH 0.5 A g^−1^	51
bamboo	1472	KOH	301	6 M KOH 0.1 A g^−1^	46
pumpkin	2968	KOH	419	6 M KOH 1 A g^−1^	52
Willow catkin	1533	KOH	298	6 M KOH 0.5 A g^−1^	53
Bamboo shoot	972	hydrothermal treatment + carbonization	412	6 M KOH 0.9 A g^−1^	This work

**Figure 8:**
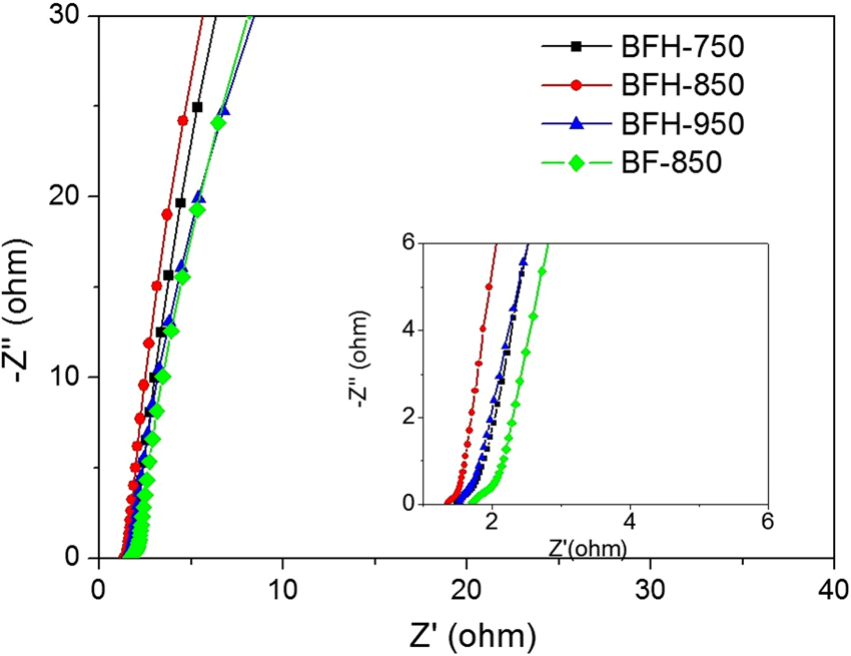
Nyquist plots of BF-850, BFH-750, BFH-850, and BFH-950 based electrode materials under the influence of an AC voltage of 5 mV.

**Figure 9:**
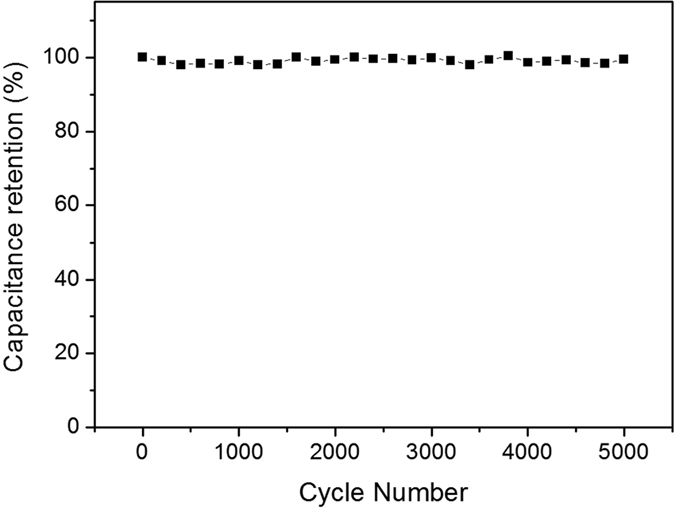
The long cycle stability of BFH-850 at a current density of 15 A g^−1^.

To further study the electrochemical performance of bamboo shoot-derived carbons, an electrochemical impedance spectroscopy (EIS) measurement was carried out and the results were shown in Fig. 8. The BHF samples showed steep linear curves in the low-frequency region, suggesting typical capacitive behavior^45^. The results revealed good accessibility of hierarchical pore for the electrolyte. The equivalent series resistances (ESR) of BFH-750, BFH-850 and BFH-950 was 1.49 Ω, 1.35 Ω and 1.50 Ω, respectively, lower than that of BF-850 (1.71 Ω). The results confirmed the best electrochemical performance of BFH-850. The long cycle stability of BFH-850 was also tested at a current density of 15 A g^−1^. As shown in Fig. 9, the relative capacitance was maintained well after 5000 cycles with 99.5% of capacitance retention, demonstrating a good cycling stability.

Based on the results above, the outstanding electrochemical performance of these bamboo shoot-derived N-doped carbons might be attributed to a synergistic factors such as hierarchically porous structure, a large surface area and nitrogen incorporation. Firstly, the hierarchical porous carbons offered a high specific surface area and abundant active sites, which ensured the rapid diffusion of ions. The mesopores might act as the buffering reservoirs, while the micropores might provide abundant adsorption sites and offer sufficient space to allow access to the electrolyte ions transportion, and thus improve the performance in supercapacitors. Secondly, the nitrogen doping of carbon might not only improve the surface wettability of the electrode materials^46^, but also enhance electronic conductivity as well as extra pseudocapacitance.

## Conclusions

In summary, a facile and sustainable approach through hydrothermal treatment and subsequent carbonization process was used to fabricate hierarchical N-doped carbon materials with nanostructures by using renewable bamboo shoots as the raw material without any templates, additional chemical activation and nitrogen source. The N-doped carbon materials were endued with a large BET surface area, hierarchically interconnected porous framework, and uniform nitrogen dopant distribution. Taking the advantages of the structure and composition, the hierarchical N-doped carbon materials displayed an outstanding electrochemical capacitive performance in KOH electrolyte and long cycling stability. The excellent performance might benefit from the synergetic effect of high BET surface area coupled with hierarchical meso/microporosity and nitrogen functionality. More importantly, the strategy put forward here also offered a good sample to utilize fully the abundant renewable resources freely available by nature to produce high-performance electrode for supercapacitors. We assume that these advanced nanostructured N-doped carbons, which could be easily produced by a low-cost and green approach in large scale, holds great promise for various practical applications like Li-ion batteries, catalysis and biosensors. Work along the applications of these nanostructured N-doped carbons as support for the heterogeneous catalysis in water system is ongoing in our lab.

## Methods

### Preparation of the bamboo shoot-derived porous carbon

Fresh bamboo shoots were obtained from Anhui Taiping Test Centre, International Centre for Bamboo and Rattan, Anhui Province, China. The bamboo shoot peels were stripped, cut into pieces and dried at 70 °C overnight. The dried bamboo shoots were then ground into powder and used as the sole material source to prepare porous N-doped carbons via hydrothermal treatment method. In a typical process, 2 g dried bamboo shoot powders were mixed with 18 mL deionized water and 0.5 mL concentrated sulfuric acid and then stirred for 30 min. Then the mixture was moved to a Teflon-lined autoclave, followed by treated at 180 °C for 5.5 h to form hydrochars. After the hydrothermal treatment, the resulting dark brown solids were collected, washed with distilled water by filtration and dried at 25 °C in vacuum for 24 h. Finally, the dried hydrochars were post-carbonized to a desired temperature (750 °C, 850 °C and 950 °C) in nitrogen gas flow and maintained for 2 h, followed by cooling to room temperature naturally under nitrogen atmosphere. For comparison, the dried bamboo shoots was calcined at 850 °C for 2 h under nitrogen atmosphere.

## Electronic supplementary material

Supplementary Information
