# Supplementary material for: A novel hierarchical porous nitrogen-doped carbon derived from bamboo shoot for high performance supercapacitor
Source: Sci Rep. 2017 Aug 4;7:7362. doi: 10.1038/s41598-017-06730-x (PMC5544758; doi:10.1038/s41598-017-06730-x)
Supplement: Supplementary file 1 — Supplementary Information [file 41598_2017_6730_MOESM1_ESM.doc]

**Electronic Supplementary Information**

**A novel hierarchical porous nitrogen-doped carbon derived from bamboo shoot for high performance supercapacitor**

Xiufang Chen^1,2,†^, Junyi Zhang^2,†^, Bo Zhang^2^, Shanmu Dong^2^, Xingcui Guo^2^, Xindong Mu^2,*^, Benhua Fei^1,*^

^1^ International Centre for Bamboo and Rattan, Beijing 100102, P.R. China. E-mail: feibenhua@icbr.ac.cn

^2^ Key Laboratory of Bio-based Materials, Qingdao Institute of Bioenergy and Bioprocess Technology, Chinese Academy of Sciences, Qingdao 266101, P.R. China. E-mail: muxd@qibebt.ac.cn

^*^ Correspondence and requests for materials should be addressed to X.M. (email: [muxd@qibebt.ac.cn](mailto:muxd@qibebt.ac.cn)) or B.F. (feibenhua@icbr.ac.cn)

† These authors contributed equally to this work.

Tel.: +86-010-84789788

Fax: +86-010-84789717

**Characterization**

The structure of carbon materials were investigated by X-ray diffraction (XRD) using a Bruker D8 Advanced X-ray diffractometer equipped with a CuKa radiation filter (λ= 1.5147 Å). The diffraction data were collected at room temperature with 2θ scan range between 5°and 80°. A field emission Hitachi S-4800 scanning electron microscope (SEM), an FEI Tecnai G2 F30 transmission electron microscopy (TEM) and an FEI Tecnai G2 F20 scanning transmission electron microscopy (STEM) were used to investigate the morphology and elemental distributions of carbon materials. The nitrogen sorption isotherms were recorded by a Micromeritics ASAP 2020 m+c sorptometer. Prior to measurements, the samples were degassed at 200 ^o^C for 8 h in vacuum and then analyzed at 77 K. The specific surface area, micropore volume and porosity distribution were estimated by the Brunauer-Emmett-Teller (BET) method, t-plot method and non-local density functional theory (NLDFT), respectively. The elemental compositions (C, N and H) of the carbon materials were measured by a Vario El elemental analyzer. The structure and surface chemical compositions of the prepared carbons were also characterized by X-ray photoelectron spectroscopy (XPS) by using a Thermo ESCALAB250 instrument. C 1s peak at 284.6 eV, originated from the possible adventitious hydrocarbon or surface hydrocarbons, was chosen for binding energy correction. The surface functional groups of carbon materials were examined by a Thermo Nicolet FTIR Spectrometer and a DXR Raman Microscope (Thermo Scientific) with a laser wavelength of 532 nm.

**Electrochemical measurements**

In a typical three-electrode system, Pt plate and saturated calomel electrode was used as counter electrode and reference electrode, respectively. 6.0 M KOH solution was used as the electrolyte. To prepare the working electrode, the N-doped carbon materials was mixed firstly with poly(vinylidene fluoride) (PVDF) and acetylene black by the weight ratio of 8:1:1, and then a certain amount of N-methyl pyrrolidone was added into the mixture to yield a homogeneous slurry. Subsequently, the slurry was loaded uniformly on a nickel foam (1 cm*1cm) and dried at 70 ^o^C for 8 h in vacuum to a constant weight, followed by pressed at 10 MPa for 5 min to obtain assembled electrodes. Cyclic voltammetry (CV) and Galvanostatic charge-discharge measurement was performed on a CHI440A electrochemical workstation at 25 ^o^C. CV curves were collected between -0.1 and 0 V at various scan rates ranging from 5 to 100 mV s-1. Galvanostatic charge-discharge tests were carried out in the potential between -1.0 and 0 V at current densities from 0.9 to 50.0 A g^-1^. Electrochemical impedance spectroscopy (EIS) was performed on a Zahner Zennium electrochemical workstation at open-circuit voltage in the frequency range from 100 mHz to 100 kHz and a 5 mV AC amplitude.

The specific capacitance (Cs) derived from the discharge curves were calculated by using the equation: Cs = I Δt/mΔV, in which I, V, t and m were the current density (A), the operating potential (V), discharge time (s) and the mass (g) of the electrode material in the three-electrode system, respectively.

**Figure S1** XRD patterns of BF-850, BFH-750, BFH-850, and BFH-950.

**Figure S2** Raman spectra of BF-850, BFH-750, BFH-850, and BFH-950.

**Figure S3** XPS survey spectrum of BFH-850.

**Figure S4** FTIR spectra of BF-850, BFH-750, BFH-850, and BFH-950.
